# Development and validation of Chronic Kidney Disease Knowledge, Attitude, and Practices (CKD-KAP) questionnaire

**DOI:** 10.3389/fmed.2022.956449

**Published:** 2022-10-11

**Authors:** Muhammad Haseeb Tariq, Syed Azhar Syed Sulaiman, Muhammad Junaid Farrukh, Khang Wen Goh, Long Chiau Ming

**Affiliations:** ^1^Department of Clinical Pharmacy, School of Pharmaceutical Sciences, Universiti Sains Malaysia, Penang, Malaysia; ^2^Advance Medical and Dental Institute, Universiti Sains Malaysia, Bertam, Penang, Malaysia; ^3^Faculty of Pharmaceutical Sciences, UCSI University, Kuala Lumpur, Malaysia; ^4^Faculty of Data Science and Information Technology, INTI International University, Nilai, N.Sembilan, Malaysia; ^5^PAP Rashidah Sa’adatul Bolkiah Institute of Health Sciences, Universiti Brunei Darussalam, Gadong, Brunei Darussalam

**Keywords:** chronic kidney disease, validation, development, KAP, renal disease

## Abstract

**Background:**

Chronic Kidney Disease (CKD) is a complex condition leading to loss of kidney function. The objective of this study was to develop and validate a Knowledge, Attitude, and Practice questionnaire on CKD (CKD-KAP) among practicing physicians in Pakistan since no validated tool was available for the said purpose.

**Methods:**

The study consisted of four phases with phase-I focusing on literature review, phase II was the actual questionnaire development phase, face and content validity was determined in phase III, and finally pilot testing was performed in phase IV to determine validity and reliability. The development phase encompassed a thorough review of literature, focus-group discussion, expert review, and evaluation. The validation phase consisted of content validity, face validity, construct validity, convergent validity, and reliability. The pilot testing was performed by studying the KAP of 100 practicing physicians in tertiary care hospitals in Pakistan. The knowledge section of the validation phase utilized Item Response Theory (IRT) analysis. The attitude and practices sections utilized Exploratory Factor Analysis (EFA) theory. The reliability analysis utilized Cronbach’s alpha and correlations.

**Results:**

The CKD-KAP questionnaire had three main sections: knowledge, attitude, and practice. During the validation, IRT analysis was performed on knowledge, which focused on the measure of the coefficient of discrimination and difficulty of the items; 40 out of 41 knowledge items have both discrimination and difficulty coefficients within an acceptable range. The EFA model was also fitted in the attitude and practices section, and scree plot and Eigenvalues suggested three and four dimensions within the attitude and practices section. The factor loading of all items was found to be acceptable except for one item in attitude which was deleted. The convergent validity demonstrated a significant association between all three sections except knowledge and practices. The reliability (internal consistency) analysis demonstrated Cronbach’s alpha values above 0.7 and significant inter-item correlation. The final model of CKD-KAP had 40 knowledge, 13 attitude, and 10 practice items with a combination of both positive as well as negative questions and statements.

**Conclusions:**

The CKD-KAP was found to be psychometrically valid and reliable, hence can be used to determine the knowledge, attitude, and practices of physicians toward chronic kidney disease.

## Background

Knowledge is a set of understandings a person has regarding any particular subject. Attitude refers to a set of beliefs, behaviors, or tending to a particular subject, while practice is a set of actions an individual takes in response to stimuli based on the understanding and tendency toward that subject (1, 2). The knowledge and attitude studies are based on a model, according to which the accumulated knowledge in a health aspect leads to changes in attitude resulting in behavior change, slowly becoming part of everyday routine practice (3). It is obvious from this model that having good knowledge is important to developing a positive attitude and good practices. Thus, the study of Knowledge Attitude Practices (KAP) is very important to identify problems, needs, and possible barriers to help to determine educational plans for medical professionals. The KAP is also suitable for assessing the performance of any already developed program (4). In modern healthcare research, KAP studies have become very significant in fields such as nutrition, disease, hygiene, and smoking prevention based on a study of community or healthcare professionals (5–9). However, most of the time, the questionnaire used to access KAP is not properly validated and thus leads to a lack of reliable knowledge, attitude, and practice scores. If a questionnaire is not validated, it might not assess the components that it should actually be measuring (10). After a thorough review of the literature, the researchers were unable to find any reliable properly validated tool for non-nephrologist physicians to access their knowledge, attitude, and practices toward chronic kidney disease. There is a need for a reliable and valid tool to assess CKD knowledge attitude and practice among physicians because it will identify the knowledge level of physicians along with their practices for the policymakers and health authorities to develop national plans for training and curriculum revisions and so on to improve the overall outcomes for CKD patients. Therefore, this study aimed to develop a questionnaire on Knowledge, Attitude, and Practice of Chronic Kidney Disease (CKD-KAP) among practicing (non-nephrologist) physicians in Pakistan.

## Materials and methods

The development and validation of the questionnaire were completed in four phases. The first phase consisted of a literature review, in which studies were identified through a comprehensive literature search of databases including PubMed, EMBASE, LILACS, Scopus, and Web of Science from the inception of these sources until January 2019. The keywords used for searching relevant articles were “knowledge,” “attitude,” “practices,” “Chronic Kidney Disease,” “management of CKD,” “Chronic Renal Failure,” and “physicians.” Boolean operators such as “AND” and “OR” were used to increase the sensitivity and specificity of the search when needed.

We identified knowledge gaps determined by previous studies and reviewed the guidelines for diagnosis and management of CKD for identification of previously explored latent variables including etiology, symptoms, diagnosis, screening, staging, dialysis, pharmacotherapy, and outcomes of CKD. A similar type of study was also reviewed but no study focusing on the development and validation of a comprehensive tool for analysis of knowledge, attitude, and practices of physicians toward CKD could be identified.

The phase II was the questionnaire development stage in which one expert from each specialty including general medicine, nephrology, clinical research, and biostatistics was included. The multidisciplinary team generated the item and its corresponding response scale for each latent variable in light of clinical management guidelines and literature review including a review of items from previously developed KAP instruments. The consensus was obtained for each item before its inclusion in the CKD-KAP, and a mixture of both truly as well as falsely articulated items were included. Then, by putting all the questions under the determined latent variables and sections, a basic form of the questionnaire was obtained. In Phase III, the developed tool was subjected to review and then content validity by a multidisciplinary team of three experts including academic researchers and clinical practitioners was determined. The face validity was performed using 10 physicians from a tertiary care hospital in Pakistan. During this phase, the referential meaning of each item was established to clarify the intended meaning of each item and the latent variables under study. The suggestions of the multidisciplinary expert team and the response received during content and face validity were incorporated into the questionnaire. In the final Phase IV, the questionnaire was administered by the researcher to a study population of 100 (non-nephrologist) physicians. The filled questionnaire was then used to determine the construct validity and reliability analysis.

**FIGURE 1:**
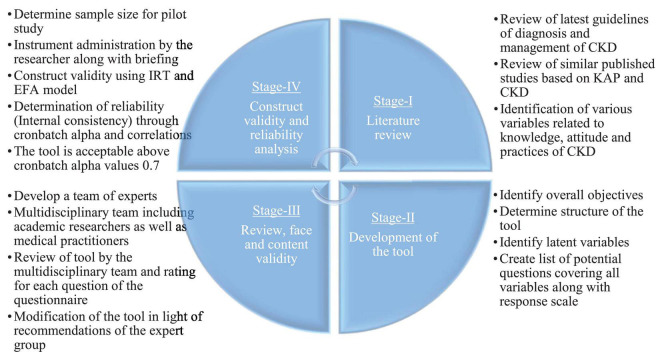
Process of development and validation of CKD-KAP questionnaire.

The initial questionnaire which was developed in Phase II contains 72 questions for knowledge, attitude, and practices. The expert multidisciplinary team including one academic researcher and two physicians recommended removing three questions from the knowledge section, one question from the attitude section, and three questions from the practices section. After the completion of phase III, a 55-scale questionnaire was obtained which was then subjected to pilot testing for construct validity and reliability analysis. The steps involved in the development and validation of questionnaire are provided in Figure 1.

### Knowledge section in CKD-KAP

Items related to general knowledge about the disease, diagnosis, risk factors, complications, and management of the disease were included in the knowledge section of CKD-KAP. Most of the questions were based on technical information related to CKD which is clearly defined by the latest KDOQI/KDIGO guidelines. A total of 42 items of knowledge having three options, that is, yes, no, and do not know with only one correct answer were developed. All the items included had sufficient difficulty levels to be answered by practicing physicians. Both positive and negative questions were added to the questionnaire. For every correct answer for an item, one point is given, while zero point is given to every incorrect answer and a higher score indicates better knowledge. Thus, the possible range for the knowledge score is 0–42 points.

### Attitude section in CKD-KAP

Items included in this section focused on the general attitudes related to CKD disease, kidney damage and outcomes, and finally the attitudes toward dialysis. A total of 15 items were developed based on the information gathered from previous studies and similar tools during the literature review phase. Subjective statements were developed and added in the initial version of CKD-KAP for each of the identified latent variables during the literature review. Both positive and negative questions were added in the attitude section. Each item was graded on a 5-point Likert scale, which includes “strongly disagree,” “disagree,” “neutral,” “agree,” and “strongly agree.” Points are given in ascending order, with one point given for the response of “strongly disagree” and five points given for the response of “strongly agree” for all the 12 positive items. Three negatively structured items were also included for which reverse scoring was applied while analysis. The scores from all individual items were added to obtain an overall score for attitude, with higher scores indicating a positive attitude. The total score ranged from a minimum of 15 to a maximum of 75 for the attitude section.

### Practice section in CKD-KAP

Items related to general practices of CKD disease, the estimation of GFR, monitoring of the disease, and complications of CKD were included in this section. A total of 13 practice items were developed on a frequency scale with a response scale having four values, that is, “never,” “seldom,” “often,” and “always” were added to the CKD-KAP questionnaire. Three negatively structured items were also developed for which reverse scoring was applied. One point is given for the lowest frequency, that is, “never” and four points were given to the highest frequency, that is, “always” for all the positively structured items in the scale. To obtain the practice score, the points from each item are added which will lead to a minimum possible score of 13 and a maximum possible score of 52. The higher score indicates better practices for CKD.

### Ethical approval

Ethical approval for this study was taken from two large tertiary care hospitals in Pakistan including Pakistan Institute of Medical Sciences (PIMS) Hospital, Islamabad, and Nawaz Sharif Kidney Hospital (NSKH), Swat. Ethical approval was granted by the Ethics Review Board of both hospitals after discussing the whole research, its importance, and future prospects. Informed consent was also obtained from all participants at the time of inclusion in the study. All methods in this study were performed in accordance with the ethical practices, guidelines, and regulations of the study institutes as well as guidelines of the declaration of Helsinki.

### Study sample

The study sample included (non-nephrologist) physicians and general practitioners working in tertiary care hospitals in Pakistan. All the physicians from the study sample were working in general medicine and they used to refer critical patients to relevant sub-specialties such as nephrology, cardiology, neurology, and so on within the hospital.

The sample size was selected based on the literature references where a sample size of 100 was considered adequate for applying the IRT, Rasch-model, and EFA model (11, 12). A sample size of 100 was required for an EFA study whenever 10–15 items were expected to have factor loadings of 0.4 (12). Based on the literature references, a sample size of 100 was selected. The questionnaire was provided to 100 physicians by the researcher and requested them to fill in the presence of the researcher to avoid any further confusion. The response rate of this study was 94% (*n* = 94) since six physicians returned the partially filled questionnaire and regardless of the reminder, they did not agree to fill the questionnaire.

### Validity

The overall validity of the CKD-KAP was analyzed by studying content validity, face validity, and construct validity.

#### Content validity

Content validity refers to the degree to which the items on a measuring scale determine the same content (13). The content validity was performed by determination of the content of CKD-KAP by an expert panel, comprising three medical professionals including one academic researcher expert in the relevant field and two senior professors of nephrology having more than 15 years of practice experience in tertiary care settings. The expert panel rated each item in CKD-KAP as “essential,” “useful,” or “not necessary,” based on its appropriateness, uncertainty, and accuracy in the questionnaire. The questionnaire was amended based on the recommendations of the expert panel.

#### Face validity

Face validity has a unique and important role to determine the perception of respondents on the appropriateness of a test (14). After content validity, the CKD-KAP was subjected to face validity by testing it among 10 physicians in a tertiary care hospital. CKD-KAP was distributed among the physicians by the researcher along with instructions on how to fill the questionnaire. All physicians were requested to complete the CKD-KAP and were stimulated to ask questions about uncertain or confusing items. These items were then clarified by trained researchers in a more comprehensive way. The questions which were still confusing even after explanation were then removed from the CKD-KAP.

#### Construct validity

Construct validity measures the underlying hypothetical concepts that the test was designed to measure (14). The construct validity was performed using Exploratory Factor Analysis (EFA) (15) and Item Response Theory (IRT) (16), since one of these models cannot fit for knowledge, attitude, and practices scale at a time.

Item Response Theory was used to fit the knowledge scale using a one-parameter logistic item response model (1P IRT Model) using STATA version 14.0. This model was selected because all knowledge items have a dichotomous scale and due to the low sample size of 100 physicians since a greater sample size is required for 2P IRT modeling. The coefficient of discrimination was determined as a single value for all the items and the coefficient of difficulty was determined for each individual item. Difficulty in the range of −3 to +3 was considered acceptable (17). Item fit was determined by the chi-square goodness-of-fit *p*-value per item and internal consistency by item-total correlation and Cronbach’s alpha.

The attitude and practices sections were fitted by using a EFA model using SPSS version 22.0 because all items in these sections have four to five response items ordinal Likert scale. The principal axis factoring extraction method, with varimax rotation, was utilized in the EFA. The items in each section were considered as a continuous response to allow estimation of the dimensionality (number of factors) of the items. To identify the number of extracted factors, eigenvalues > 1.0, parallel analysis, and scree plot inspection were utilized. Factor loadings > 0.4 were considered acceptable. The number of extracted factors having eigenvalues > 1.0 was studied for internal consistency for all the included items using Cronbach’s alpha.

#### Convergent validity

Convergent validity refers to a concept that shows a high correlation with a theoretically similar concept. Convergent validity of a particular section (i.e., knowledge, attitude, or practices) of the survey instrument could be established if items of a section correlate strongly with the same section of other instruments developed for the same purpose. In the absence of any other valid and reliable tool for accessing the KAP of physicians regarding CKD, the exact convergent validity could not be performed. Therefore, a circular approach was adopted in which correlations between different sections of the same instrument were determined. This approach is also used by various studies conducted for similar purposes. Convergent validity, therefore, was performed by studying the correlation between total knowledge, attitude, and practices score. Correlation coefficient “*r*” value and *p*-value for Pearson correlation were studied.

### Reliability

The reliability analysis was performed using internal consistency which is one of the commonly employed approaches to determine the reliability of any instrument (18). Internal consistency and reliability are aimed to analyze the similarity and closeness in terms of the response of items or a set of items to each other within a domain.

The reliability analysis through internal consistency was determined using the inter-item correlation. Since the attitude and practice sections of the CKD-KAP contain continuous data, the reliability coefficients (Cronbach’s alpha) were also determined. Drasgow et al. reported that the reliability coefficient (Cronbach’s alpha) value of 0.6 is suitable and acceptable for exploratory research (19). However, the more conservative cut-off Cronbach’s alpha value of 0.7 was selected for this study.

### Statistical analysis

Data were analyzed using the IBM Statistical Package for Social Sciences (SPSS) for Windows Version 22.0 software (20). All assumptions to conduct the EFA were met during the study and analysis. Initially, Bartlett’s test of sphericity was found to be significant (*p* < 0.0001). Second, the requirements for overall Measure of Sampling Adequacy (MSA) were met, with a value of 0.765 for attitude and 0.689 for the practice section of CKD-KAP. All correlations were considered significant at a statistical level of *p* < 0.05 and Cronbach’s alpha value >0.7 were considered acceptable for internal consistency reliability.

## Results

### Demographic characteristics

Table 1 shows the demographic of the physicians under the study. The questionnaire was administered to 100 physicians out of which 94 returned the completed questionnaire with a response rate of 96%. This study thus involved a total of 94 physicians including 56 (59.6%) men and 38 (40.4%) women. About 62 (66%) physicians were less than 30 years of age, 26 (27.7%) were 31–45 years, and 6 (6.4%) were 46–60 years of age. Our study included physicians from different practice settings, 80 (85.1%) physicians were practicing only in public tertiary care hospitals, while 14 (14.9%) were practicing in both public as well as private clinics. About 58 (61.7%) physicians have graduated in medicine and surgery MBBS or MD, 30 (31.9%) were post-graduate trainees, and 6 (6.4%) were consultants after completing their specialization. The consultant is a senior physician who has completed all their specialist training and been placed on the specialist register in their respective specialty. All the physicians under study have a different level of clinical experience, 46 (48.9%) physicians have only done a house job which is a mandatory 1-year clinical practice to be performed under the supervision of senior professors in tertiary care hospitals at the end of the graduate degree, 22 (23.4%) physicians have less than 5 years of experience, and 26 (27.7%) physicians have more than 5 years of clinical experience.

**TABLE 1:** Demographics of physicians under study.

Demographic variables		Frequency (%)
Gender	Male	56 (59.6)
	Female	38 (40.4)
Age	<30 years	62 (66)
	31–45 years	26 (27.7)
	46-60 years	6 (6.4)
Practice setting	Public	80 (85.1)
	Both public and private	14 (14.9)
Level of education	Graduation (MBBS) only	58 (61.7)
	Post graduate trainee	30 (31.9)
	Consultant	6 (6.4)
Clinical experience	House job	46 (48.9)
	<5 years	22 (23.4)
	>5 years	26 (27.7)

### Content validity

For the knowledge section of the CKD-KAP questionnaire, a total of three items were not recommended by the expert group and were removed. Based on the comments provided by the expert panel, these items were not related to knowledge of CKD and repetitive of similar items. For the attitude section, one item each was deleted because the item was not actually related to the attitude of physicians. From the practices section, three items were not recommended by the expert team and were deleted since they were controversial in the literature review. The process of content validity retained 41 knowledge items, 14 attitude items, and 10 practice items making up a total of 65-item CKD-KAP questionnaire.

### Face validity

After content validity, the revised version of the CKD-KAP was used to determine face validity. Most items in the CKD-KAP were fully understood by the physicians, except for terms such as the MDRD equation which was later on explained to them and used in full form in the questionnaire.

### Construct validity

For construct validity, different models were used for the knowledge, attitude, and practices sections. One-parameter logistic item response model (1P IRT Model) of IRT was used to fit the knowledge scale. A single value of the coefficient of discrimination “*a*” was determined for all the items, while the coefficient of difficulty “*b*” was determined for each individual item.

The coefficient of discrimination value falls in acceptable criteria (0.30–2.5), with a combined value of 1.031 and with a low value of standard error, that is, 0.09. Out of 41 knowledge items, 40 items met the acceptable range for the coefficient of difficulty “*b*” (−3.0 to 3.0). Only one item has a coefficient of difficulty value above 3.0 and was removed from the questionnaire. The item-to-total correlations (ITC) for each item were above the recommended cut-off (0.20) (21). The performance of each item in the knowledge section of the CKD-KAP instrument was relatively good. The results of the IRT analysis are provided in Table 2.

**TABLE 2:** Results of the IRT analysis in the knowledge section of the validation study.

#	Item of knowledge scale	Item-total correlation	*b* (SE)	*P*
1	CKD is defined as abnormalities of kidney structure or function, present for >3 months, with implications for health	0.389	−2.43 (0.406)	<0.001
2	The KDOQI guidelines have classified CKD based on GFR values in 5 classes (G1–G5)	0.866	2.69 (0.445)	<0.001
3	The KDOQI guidelines have classified CKD based on albuminuria value in 3 classes (A1–A3)	0.521	−0.05 (0.245)	0.829
4	According to the 2017 ACC/AHA guidelines the target blood pressure in CKD patients should be <130/80 mmHg and those with blood pressure >130 mmHg will be classified as hypertensive	0.706	−1.02 (0.273)	<0.001
5	Is eGFR a better way of assessing decline in kidney function than elevated serum creatinine alone?	0.721	−1.02 (0.273)	<0.001
6	Can age related reduction in eGFR without kidney disease lead to low eGFR with normal serum creatinine, normal urine analysis and normal USG?	0.599	−0.680 (0.25)	0.008
7	Cockroft-gault equation is a better tool to estimate GFR than by MDRD equation	0.823	2.02 (0.355)	<0.001
8	The KDIGO 2012 guidelines recommended classifying CKD based on cause, GFR category and albuminuria category	0.791	0.254 (0.247)	0.303
9	Following are the risk factors which should be considered while predicting the CKD prognosis			
	(a) Elevated blood pressure	0.454	−2.69 (0.44)	<0.001
	(b) Hyperglycemia	0.572	−1.53 (0.308)	<0.001
	(c) Dyslipidemia	0.815	−0.466 (0.251)	0.063
	(d) History of cardiovascular disease	0.577	−1.84 (0.33)	<0.001
	(e) Chronic use of NSAIDs, lithium, cyclosporine	0.350	−2.43 (0.406)	<0.001
	(f) Glomerulonephritis	0.443	-2.434 (0.406)	<0.001
10	Following are the complications for which every CKD patient should be continuously monitored			
	(a) Anemia	0.471	−2.434 (0.406)	<0.001
	(b) Metabolic bone disease	0.484	−1.398 (0.297)	<0.001
	(c) Hyperkalemia	0.256	−2.215 (0.538)	<0.001
	(d) Acidosis	0.430	−2.213 (0.377)	<0.001
	(e) Edema	0.576	−2.434 (0.406)	<0.001
	(f) Acute Kidney Injury	0.584	−1.267 (0.288)	<0.001
11	ACE inhibitors are the first line drugs in the management of CKD in both diabetic and non-diabetic patients	0.644	−0.680 (0.257)	0.008
12	All patients of CKD should be considered at high risk for developing Acute Kidney Injury (AKI)	0.546	−1.267 (0.288)	<0.001
13	High protein diet should be administered to all CKD patients at risk of Acute Kidney Injury (AKI)	0.343	−0.680 (0.257)	0.008
14	Guidelines recommend use of isotonic crystalloids fluids in CKD patients with AKI to keep the hydration status	0.689	0.151 (0.246)	0.538
15	Dialysis should be initiated in CKD patients with AKI with abrupt changes in electrolytes and fluid	0.461	−0.680 (0.257)	0.008
16	Diuretics are recommended to improve kidney function in CKD patients with AKI	0.717	1.845 (0.337)	<0.001
17	Anticoagulation therapy with enoxaparin or unfractionated heparin is recommended in AKI patients on dialysis (not at risk of bleeding)	0.681	−0.361 (0.248)	0.146
18	Iron therapy is recommended in CKD patients with anemia	0.492	−1.267 (0.288)	<0.001
19	Erythropoietin therapy is not recommended at Hb > 10 g/dl	0.323	−0.904 (0.267)	0.001
20	IV Iron dextran should be continued in CKD patients with anemia having systemic infection	0.773	2.02 (0.355)	<0.001
21	Phosphate lowering therapy with phosphate binders is recommended in CKD patients at risk of mineral and bone disorders	0.557	−1.267 (0.288)	<0.001
22	The dose of calcium based phosphate binders should be restricted in G3a-G5 stage CKD patients	0.781	0.049 (0.245)	0.841
23	In CKD G5 stage patients with hyperparathyroidism calcitriol is not recommended	0.693	1.685 (0.322)	<0.001
24	KDOQI guidelines for dialysis have recommended that initiating dialysis on stage 4 patients with GFR <30ml/min may yield better clinical outcomes and low mortality rate	0.693	0.677 (0.258)	0.009
25	Anticonvulsant drugs valproic acid is dialyzable and thus require additional dose after dialysis	0.788	2.698 (0.445)	<0.001
26	Loading doses do not needs adjustments in CKD patients	0.686	0.151 (0.246)	0.538
27	Reduction in dose without changing the dosing interval may be associated with LOWER risk of toxicities	0.611	2.02 (0.355)	<0.001
28	Lengthening the dosing interval without changing the dose is associated with higher risk of subtherapeutic drug concentrations	0.576	−0.466 (0.251)	0.063
29	ACE inhibitors should be discontinued if the serum creatinine rise by more than 30%	0.615	−0.572 (0.254)	0.024
30	Metformin can be administered to stage 5 CKD patients with GFR < 15ml/min	0.733	1.139 (0.280)	<0.001
*a* (SE)		1.031 (0.09)		
Cronbach’s alpha		0.963		

The EFA model was applied to the attitude and practices sections. The Kaiser–Meyer–Olkin (KMO) measure of sampling adequacy was 0.765 which is in the acceptable range and shows the adequacy of the sample for fitting the EFA model. The model was significantly fit as demonstrated by Bartlett’s test of sphericity (*p* < 0.001). The results of test statistics of the EFA model are given in Table 3.

**TABLE 3:** Results of the test statistics for EFA analysis in the attitude section of the validation study.

Kaiser–Meyer–Olkin measure of sampling adequacy.		0.765
Bartlett’s test of sphericity	Approx. chi-square	583.941
	*p* value	0.000

Table 4 shows the results of factor analysis for the attitude sections in the CKD-KAP instrument. For the attitude section, in the EFA analysis, the scree plot and eigenvalue (>1.0) revealed three dimensions including general attitudes toward CKD, kidney damage and outcomes, and attitudes toward dialysis. In the final attitude model, three dimensions were identified with 14 items. The EFA factor loading values for all the items in each dimension was above the standard value of 0.49. The inter-item reliability in each dimension was measured in terms of Cronbach’s alpha and based on those values, one item was deleted since it was affecting the overall value of Cronbach’s alpha and thus a final attitude section containing 13 items was left. The Cronbach’s alpha values for dimension 1, 2, and 3 were 0.822, 0.838, and 0.768 which all lies in the acceptable range (>0.7).

**TABLE 4:** Results of the EFA analysis in the attitude section of the validation study.

Factor	Eigen value	Item	EFA λ	Reliability^a^
General CKD	4.647	Dose of all renally excreted drugs should not be adjusted to prevent toxicities in CKD patients	0.774	0.822
		Every CKD patient on dialysis should be regularly screened for hepatitis	0.750	
		Prescribing in line with the latest guidelines can improve clinical outcomes	0.712	
		Early diagnosis of CKD can prevent all-cause mortality	0.690	
		Physicians should study the latest clinical guidelines to provide better clinical services.	0.678	
		Clinical pharmacist can help improve clinical outcomes and reduce toxicities in CKD patients	0.611	
Kidney damage and outcomes	2.630	Providing dietary counseling to CKD patients is not required	0.915	0.838
		Every CKD patient should be considered at high risk of acute kidney injury	0.764	
		Drugs cannot cause kidney damage	0.761	
		More research is required to determine factors affecting clinical outcomes in CKD patients in Pakistan	0.756	
		CKD patients should not administer herbal and alternate medicine	0.628	
Dialysis	1.131	Dialysis facilities for hepatitis C should be kept separate	0.799	0.768
		Drug therapy of CKD patients should be reviewed after initiation of dialysis	0.712	

λ, Factor loading. ^*a*^Cronbach’s alpha.

The EFA model was also applied to the practices section and the KMO measure of sampling adequacy was 0.689 which falls in the acceptable range and shows that the sample size for this model was adequate. The model was significantly fit as demonstrated by Bartlett’s test of sphericity (*p* < 0.001). The results of test statistics of the EFA model are given in Table 5.

**TABLE 5:** Results of the test statistics for EFA analysis in the practices section of the validation study.

Kaiser–Meyer–Olkin measure of sampling adequacy.		0.689
Bartlett’s test of sphericity	Approx. chi-square	180.630
	*p* value	0.000

Table 6 shows the results of factor analysis for the practices section in the CKD-KAP instrument. The EFA analysis, in the practices section, revealed four dimensions through the scree plot and eigenvalue (>1.0) including general practices toward CKD, monitoring of CKD, measurement of GFR, and practices toward complications of CKD. The EFA factor loading values for all the items in each dimension was above the standard value of 0.49. The inter-item reliability in each dimension was measured in terms of Cronbach’s alpha and its values for dimensions 1, 2, 3, and 4 were 0.750, 0.778, 0.763, and 0.839 which all lie in the acceptable range (>0.7).

**TABLE 6:** Results of the EFA analysis in the practices section of the validation study.

Factor	Eigen value	Item	EFA λ	Reliability
General CKD	3.070	Do you refer your CKD patients to nephrologist at stage G5?	0.765	0.750
		Do you use serum creatinine to adjust medication doses in CKD patients?	0.723	
		Do you review the drug therapy of your CKD patients for potential Drug Related Problems (DRP’s) and interactions?	0.699	
		Do you provide dietary counseling to your CKD patients?	0.661	
Monitoring of CKD	1.218	Do you routinely measure urine protein (albumin) in your CKD patients?	0.654	0.778
		Do you monitor Iron, RBC and hemoglobin of your CKD patients for anemia after every 3 months?	0.484	
Measurement of GFR	1.145	Do you use MDRD equation for calculating GFR from serum creatinine?	0.785	0.763
		Do you diagnose and stage your CKD patients based on serum creatinine instead of GFR values?	0.613	
Complications of CKD	1.024	Do you recommend your CKD patients to undergo Bone Mineral Density (BMD) scan for determining osteoporosis?	0.833	0.839
		Do you routinely monitor calcium, phosphorous and PTH levels in CKD patients?	0.699	

After conducting EFA, the convergent validity of the CKD-KAP was determined. Convergent validity was determined by correlating knowledge, attitude, and practice on physicians which showed significant associations between knowledge and practices. The possible reason behind such poor correlation between knowledge and practices can be due to the lack of adequate training facilities for doctors where they can practice their theoretical knowledge, but instead, they focus more on following the senior consultants and professors regardless of having good knowledge. The correlations between knowledge and attitude (*r* = 0.460, *p* = 0.000), knowledge and practice (*r* = 0.053, *p* = 0.609), as well as attitude and practice (*r* = 0.235, *p* = 0.023) are mentioned in Table 7.

**TABLE 7:** Results of the convergent validity of the CKD-KAP questionnaire.

Parameters	Knowledge	Attitude	Practice
Knowledge	1.00		
Attitude	0.460	1.00	
Practice	0.053	0.235	1.00

### Reliability

Table 2 shows the reliability results for the knowledge while Tables 8, 9 show reliability results for attitude and practice sections of the CKD-KAP instrument. The reliability coefficient value in terms of Cronbach’s alpha for the knowledge of CKD-KAP is 0.963. In the attitude section, Cronbach’s alpha for the model is 0.829, while in the practice section, Cronbach’s alpha for the model is 0.756. All dimensions in the attitude and practice sections of the KAP-HLQ have fulfilled the minimum internal consistency reliability of at least 0.7 for such exploratory research (19).

**TABLE 8:** Results of the reliability (internal consistency) analysis in the attitude section of the validation study.

Cronbach’s alpha								0.829					
	A1	A2	A3	A4	A5	A6	A7	A8	A9	A10	A11	A12	A13
A1	1.000												
A2	0.599	1.000											
A3	0.530	0.344	1.000										
A4	0.462	0.467	0.354	1.000									
A5	0.642	0.608	0.413	0.338	1.000								
A6	0.506	0.335	0.550	0.251	0.383	1.000							
A7	0.218	0.139	-0.130	0.347	0.230	-0.036	1.000						
A8	0.225	0.119	0.096	0.299	0.268	0.067	0.617	1.000					
A9	0.160	0.115	-0.143	0.026	0.217	0.064	0.723	0.508	1.000				
A10	0.369	0.270	0.104	0.383	0.463	0.226	0.660	0.606	0.468	1.000			
A11	0.031	0.191	-0.087	0.286	0.119	0.023	0.496	0.397	0.298	0.360	1.000		
A12	0.448	0.276	0.354	0.219	0.410	0.305	0.050	0.103	0.094	-0.001	0.059	1.000	
A13	0.508	0.314	0.382	0.294	0.452	0.359	0.203	0.204	0.292	0.209	0.157	0.626	1.000

**TABLE 9:** Results of the reliability (internal consistency) analysis in the practices section of the validation study.

Cronbach’s alpha score						0.756				
	P1	P2	P3	P4	P5	P6	P7	P8	P9	P10
P1	1.000									
P2	0.109	1.000								
P3	0.221	0.216	1.000							
P4	0.067	0.576	0.121	1.000						
P5	0.166	0.453	0.170	0.355	1.000					
P6	0.227	0.136	0.227	0.090	0.153	1.000				
P7	0.618	0.088	0.217	0.142	0.296	0.266	1.000			
P8	0.156	0.432	0.145	0.416	0.334	0.046	0.199	1.000		
P9	0.108	0.172	0.191	0.141	0.311	0.725	0.250	0.000	1.000	
P10	0.159	0.258	0.643	0.212	0.311	0.159	0.216	0.096	0.132	1.000

In the attitude section of CKD-KAP, all items were found to be significantly correlated with each other, with *r*-values ranging from low to moderate (0.116–0.629). For the practice section, only four items show weak correlation while all other correlations were found to be significant ranging from low to moderate (0.116–0.629).

### Final model of CKD-KAP

The final model of the knowledge section of CKD-KAP has a total of 40 items. This will lead to a minimum possible score of 0 points and a maximum possible score of 40 points. As items of knowledge on CKD measures known facts and not abstract variables and it was studied through the IRT model, therefore, it was not further divided into domains. The knowledge section of CKD-KAP is therefore reported by total score, with a higher score indicating better knowledge of CKD. The authors consider that practicing physician should at least have 50% knowledge which corresponds to a knowledge score of 20. However, for studying the knowledge of physicians from a particular country, the mean knowledge score of the physicians could also be taken as a benchmark for good knowledge, that is, physicians having a higher score than the mean may be considered to have good knowledge and those having lower knowledge score than the mean knowledge may be considered as having poor knowledge.

A total of 13 items were retained in the attitude section of the CKD-KAP instrument. Attitude regarding CKD can be assessed by total score as well as a score within each of the three domains. The minimum possible overall attitude score can be 13 while the maximum score can be 65. A higher score will indicate a positive attitude. Based on the EFA model, the attitude section was further divided into three domains each containing six, five, and two items. For the first domain general attitudes toward CKD, the minimum possible score can be 6 while the maximum possible score can be 30. For the second domain attitudes toward kidney damage and outcomes, the minimum possible score can be 5 while the maximum possible score can be 25. The third domain attitudes toward dialysis can have a minimum score of 2 while a maximum score of 10. Just like with the overall score, a higher score in each domain indicate positive attitudes. In the attitude section, reverse scoring applies to three items which are highlighted by a * mark in the CKD-KAP questionnaire in the Supplementary file.

For the practice section of the CKD-KAP, 10 items that fit in the instrument were retained. Practice on CKD can be assessed by total practice score and by dimension score. The possible minimum total practice score is 10 and the possible maximum total practice score is 40 points. Reverse scoring applies to three negatively constructed items which are identified by the * sign in the CKD-KAP questionnaire in the Supplementary file. Since after the EFA model, the practices section was divided into four domains, and therefore to calculate practices in each domain, the minimum possible score in first domain, that is, general practices toward CKD is 4 and the maximum possible score is 16, while the minimum possible score in each of the 2nd, 3rd, and 4th domain is 2 while the maximum score in these domains is 8. The higher score in the practice section indicates better practices toward CKD.

## Discussion

The CKD-KAP is an important assessment tool since it has been developed and tailored to the physician’s aspects in their everyday clinical practice. During the validation phase, it has been tested for validity including content validity, face validity, construct validity, and convergent validity and reliability (internal consistency).

For content validity, a consensus was met among the expert group in reviewing the CKD-KAP based on the contents of all modules. It is essential to match all the items with the module as it is meant to evaluate the respective module (22, 23). Our study also included a review group of three experts for content validation for adequate inter-rater agreement (13). The CKD-KAP has three modules, that is, knowledge, attitude, and practices, which are further split into domains within each module. Similar procedures were applied by other researchers (24, 25).

Face validity is an important step in validity analysis, especially for tools that are developed for a specific population since the comprehensibility of items by the relevant target group is important (26). This significance of face validity in determining the appropriateness of an instrument for a specific target group has already been identified and defined (27). The majority of items in the CKD-KAP were well understood by the physicians, with an exception of one terminology which was later on used in full form in the questionnaire.

For construct validity, IRT was used to fit the knowledge scale using the one-parameter logistic item response model (1P IRT Model). The value of the coefficient of discrimination “*a*,” coefficient of difficulty “*b*,” and item-to-total correlations were all within the acceptable range indicating the good performance of the knowledge section of this instrument (28). The construct validity of the attitude and practices section was determined using the EFA model, and all attitude and practices items presented good factor loadings > 0.6 in attitude and > 0.4 in the practices section (15). All the parameters of the models such as KMO measure of sampling adequacy and Bartlett’s test of sphericity demonstrated result in an acceptable range. The study of scree plot and eigenvalue further split the attitude and practice module into three and four domains, respectively. These domains were then named based on the shared concept by all items grouped in each domain (29). Similar values of the factor loading, sampling adequacy, and test of sphericity were also obtained in another study focused on CKD in which the respondents were patients instead of physicians (30, 31).

Convergent validity was performed to determine the correlation between knowledge, attitude, and practices of the physicians toward CKD. The significant correlations during this analysis support the definition of convergent validity as it suggests a high correlation with a theoretically similar concept. Convergent validity demonstrated significant associations among all the parameters. On the other hand, we observed a low correlation between knowledge and practice probably due to a lack of adequate training facilities. Usually in Pakistan, due to a lack of national guidelines and health services regulation at the national level, the prescriptions are not monitored at any level and thus it leads to the development of poor practices among physicians. Furthermore, the young undertraining physicians also rely more on the lectures of senior professors and there is very little concept of the study of evidence-based latest clinical guidelines. Such kind of training environment can lead to the development of poor practices among physicians regardless of having good theoretical knowledge. Reliability (internal consistency) of the CKD-KAP also demonstrated correlation through Cronbach’s alpha in the acceptable range with all correlation values above 0.7 indicating a strong correlation between different parameters within a domain. The reliability scores obtained for CKD-KAP were comparable to already available tools for various other diseases and as per the recommended guidelines to validate the study tools (32).

There are some limitations and recommendations of the study that should be addressed. Due to the comprehensiveness of the study tool, a large number of items were included in the CKD-KAP which may sometimes take more than 10 min to fill the questionnaire. However, since a group of experts performed the content validity, that’s why the tool has been professionally developed to access the knowledge since it contains a lot of items that require in-depth overview of the disease and strong practice skills. The small sample size and younger age of most participating physicians in this study are also acknowledged as a limitation to the generalizability of the study tool. Another limitation of our study includes the content validity which is performed using a single group of experts. The content validity would have been much stronger if we had multiple groups of expert panels; therefore, blind assessments were carried out and the results were pooled to look for agreements across the expert groups. The use of the 1P IRT Model is also regarded as a limitation of the study since the use of the 3P IRT model which requires a large sample size could be a better measure of validity. Since no validated tool was already available for accessing the KAP of physicians regarding CKD, the exact approach of convergent validity could not be utilized, and instead, a circular approach was used. This could also be considered a minor limitation of the study. This instrument was validated among physicians in Pakistan and can be applied to physicians worldwide, especially in countries that have similar medical teaching and practice settings. However, for other countries, researchers are recommended to modify and validate the CKD-KAP among physicians of that country.

## Conclusion

Based on the IRT and factor analysis, the CKD-KAP questionnaire was found to be psychometrically valid and reliable. Hence, the CKD-KAP may be used to determine the knowledge, attitude, and practices of physicians (non-nephrologists) toward chronic kidney disease and can help policymakers devise changes in continuous medical education programs to improve the knowledge and practices of physicians.

## Data availability statement

The original contributions presented in this study are included in the article/Supplementary material, further inquiries can be directed to the corresponding authors.

## Ethics statement

The studies involving human participants were reviewed and approved by Pakistan Institute of Medical Sciences (PIMS) Hospital, Islamabad, and Nawaz Sharif Kidney Hospital (NSKH), Swat. The patients/participants provided their written informed consent to participate in this study.

## Author contributions

MT: literature review and data collection. MF: data analysis and write up. SS: supervision and review. KG: analysis. LM: review. All authors contributed to the article and approved the submitted version.

## Abbreviations

CKD: Chronic Kidney Disease
KAP: Knowledge attitude and practice
IRT: Item Response Theory
EFA: Exploratory Factor Analysis
KDOQI: kidney disease outcomes quality initiative
KDIGO: kidney disease improving global outcomes
GFR: glomerular filtration rate
MBBS: bachelor in medicine bachelor in surgery
MD: doctor of medicine
CFA: confirmatory factor analysis
RBC: red blood cell
BMD: bone mineral density
PTH: parathyroid hormone.
