# Supplementary material for: Development and validation of Chronic Kidney Disease Knowledge, Attitude, and Practices (CKD-KAP) questionnaire
Source: Front Med (Lausanne). 2022 Oct 11;9:956449. doi: 10.3389/fmed.2022.956449 (PMC9592727; doi:10.3389/fmed.2022.956449)
Supplement: Supplementary file 1 [file Data_Sheet_1.PDF]

Chronic Kidney Disease Knowledge, Attitude and Practices (CKD-KAP) questionnaire

Hospital Name: ____________________ Country State / Province: ____________________

PART-I DEMOGRAPHICS

Age: ☐ Years
Clinical Experience ☐ Years
Gender: ☐ Male ☐ Female
Practice setting: ☐ Public ☐ Private ☐ Both
Level of Education: ☐ MBBS ☐ PG Trainee ☐ Consultant
Any special training of Nephrology/ CKD: ☐ Yes ☐ No
Have you read KDOQI / KDIGO guidelines for management of CKD? ☐ Yes ☐ No
Source of Information (For CKD) ☐ Books and guidelines ☐ Internet ☐ Special training ☐ CME activity

PART-II KNOWLEDGE ABOUT CKD

Q#	Particulars	Yes	No	Don't know
1.	CKD is defined as abnormalities of kidney structure or function, present for >3 months, with implications for health			
2.	The KDOQI guidelines have classified CKD based on GFR values in 5 classes (G1 to G5)			
3.	The KDOQI guidelines have classified CKD based on albuminuria value in 3 classes (A1 to A3)			
4.	According to the 2017 ACC/AHA guidelines the target blood pressure in CKD patients should be <130/80 mmHg and those with blood pressure >130 mmHg will be classified as hypertensive			
5.	Is eGFR a better way of assessing decline in kidney function than elevated serum creatinine alone?			
6.	Can age related reduction in eGFR without kidney disease lead to low eGFR with normal serum creatinine, normal urine analysis and normal USG?			
7.	Cockroft-gault equation is a better tool to estimate GFR than by MDRD equation*			
8.	The KDIGO 2012 guidelines recommended classifying CKD based on cause, GFR category and albuminuria category			
9.	**Following are the risk factors which should be considered while predicting the CKD prognosis**			
	a) Elevated blood pressure			
	b) Hyperglycemia			
	c) Dyslipidemia			
	d) History of cardiovascular disease			
	e) Chronic use of NSAIDs, lithium, cyclosporine			
	f) Glomerulonephritis			
10.	**Following are the complications for which every CKD patient should be continuously monitored**			
	a) Anemia			
	b) Metabolic bone disease			
	c) Hyperkalemia			
	d) Acidosis			
	e) Edema			
	f) Acute Kidney Injury			

11.	ACE inhibitors are the first line drugs in the management of CKD in both diabetic and non-diabetic patients			
12.	All patients of CKD should be considered at high risk for developing Acute Kidney Injury (AKI)			
13.	High protein diet should be administered to all CKD patients at risk of Acute Kidney Injury (AKI)*			
14.	Guidelines recommend use of isotonic crystalloids fluids in CKD patients with AKI to keep the hydration status			
15.	Dialysis should be initiated in CKD patients with AKI with abrupt changes in electrolytes and fluid			
16.	Diuretics are recommended to improve kidney function in CKD patients with AKI*			
17.	Anticoagulation therapy with enoxaparin or unfractionated heparin is recommended in AKI patients on dialysis (not at risk of bleeding)			
18.	Iron therapy is recommended in CKD patients with anemia			
19.	Erythropoietin therapy is not recommended at Hb > 10 g/dl			
20.	IV Iron dextran should be continued in CKD patients with anemia having systemic infection*			
21.	Phosphate lowering therapy with phosphate binders is recommended in CKD patients at risk of mineral and bone disorders			
22.	The dose of calcium based phosphate binders should be restricted in G3a-G5 stage CKD patients			
23.	In CKD G5 stage patients with hyperparathyroidism calcitriol is not recommended*			
24.	KDOQI guidelines for dialysis have recommended that initiating dialysis on stage 4 patients with GFR <30ml/min may yield better clinical outcomes and low mortality rate			
25.	Anticonvulsant drugs valproic acid is dialyzable and thus require additional dose after dialysis*			
26.	Loading doses do not needs adjustments in CKD patients			
27.	Reduction in dose without changing the dosing interval may be associated with LOWER risk of toxicities*			
28.	Lengthening the dosing interval without changing the dose is associated with higher risk of subtherapeutic drug concentrations			
29.	ACE inhibitors should be discontinued if the serum creatinine rise by more than 30%			
30.	Metformin can be administered to stage 5 CKD patients with GFR < 15ml/min*			

PART-III ATTITUDE TOWARDS CKD

Answer the following questions according to 5-point Likert Scale defined below

Strongly disagree	Disagree	Neutral	Agree	Strongly agree
**1**	**2**	**3**	**4**	**5**

Q #	**Statement**	**1**	**2**	**3**	**4**	**5**
**General attitudes towards CKD**						
1.	Dose of all renally excreted drugs should not be adjusted to prevent toxicities in CKD patients*					
2.	Every CKD patient on dialysis should be regularly screened for hepatitis					
3.	Prescribing in line with the latest guidelines can improve clinical outcomes					
4.	Early diagnosis of CKD can prevent all-cause mortality					
5.	Physicians should study the latest clinical guidelines to provide better clinical services.					
6.	Clinical pharmacist can help improve clinical outcomes and reduce toxicities in CKD patients					
**Kidney damage and outcomes**						
7.	Providing dietary counseling to CKD patients is not required*					
8.	Every CKD patient should be considered at high risk of acute kidney injury					
9.	Drugs cannot cause kidney damage*					
10.	More research is required to determine factors affecting clinical outcomes in CKD patients in Pakistan					
11.	CKD patients should not administer herbal and alternate medicine					
**Dialysis**						
12.	Dialysis facilities for hepatitis C should be kept separate					
13.	Drug therapy of CKD patients should be reviewed after initiation of dialysis					

PART-IV PRACTICES ABOUT CKD

Answer the following questions according to 4-point Likert Scale defined below

Never	Seldom	Often	Always
**1**	**2**	**3**	**4**

Q #	Statement	1	2	3	4
	**General practices for CKD**				
1.	Do you refer your CKD patients to nephrologist at stage G5?*				
2.	Do you use serum creatinine to adjust medication doses in CKD patients?*				
3.	Do you review the drug therapy of your CKD patients for potential Drug Related Problems (DRP's) and interactions?				
4.	Do you provide dietary counseling to your CKD patients?				
	**Monitoring of CKD**				
5.	Do you routinely measure urine protein (albumin) in your CKD patients?				
6.	Do you monitor Iron, RBC and hemoglobin of your CKD patients for anemia after every 3 months?				
	**Measurement of GFR**				
7.	Do you use MDRD equation for calculating GFR from serum creatinine?				
8.	Do you diagnose and stage your CKD patients based on serum creatinine instead of GFR values?*				
	**Complications of CKD**				
9.	Do you recommend your CKD patients to undergo Bone Mineral Density (BMD) scan for determining osteoporosis?				
10.	Do you routinely monitor calcium, phosphorous and PTH levels in CKD patients?				

THANK YOU
